# Effect of leaf phenology and morphology on the coordination between stomatal and minor vein densities

**DOI:** 10.3389/fpls.2023.1051692

**Published:** 2023-07-26

**Authors:** Wanli Zhao, Peili Fu, Qinggong Mao, Guolan Liu, Yuanqiu Li, Jiangbao Xia, Ping Zhao

**Affiliations:** ^1^ Shandong Key Laboratory of Eco-Environmental Science for Yellow River Delta, Binzhou University, Binzhou, Shandong, China; ^2^ Key Laboratory of Vegetation Restoration and Management of Degraded Ecosystems, South China Botanical Garden, Chinese Academy of Sciences, Guangzhou, Guangdong, China; ^3^ CAS Key Laboratory of Tropical Forest Ecology, Xishuangbanna Tropical Botanical Garden, Chinese Academy of Sciences, Jinghong, Yunnan, China; ^4^ Ailaoshan Station of Subtropical Forest Ecosystem Studies, Xishuangbanna Tropical Botanical Garden, Chinese Academy of Sciences, Jingdong, Yunnan, China; ^5^ Guangdong Shimentai National Nature Reserve, Guangdong Forestry Administration, Qingyuan, Guangdong, China

**Keywords:** water physiology, deciduous trees, compound leaf, stomatal density, minor vein density

## Abstract

Leaf phenology (evergreen vs. deciduous) and morphology (simple vs. compound) are known to be related to water use strategies in tree species and critical adaptation to certain climatic conditions. However, the effect of these two traits and their interactions on the coordination between minor vein density (MVD) and stomatal density (SD) remains unclear. In this study, we examined the leaves of 108 tree species from plots in a primary subtropical forest in southern China, including tree species with different leaf morphologies and phenologies. We assessed nine leaf water-related functional traits for all species, including MVD, SD, leaf area (LA), minor vein thickness (MVT), and stomatal length (SL). The results showed no significant differences in mean LA and SD between either functional group (simple vs. compound and evergreen vs. deciduous). However, deciduous trees displayed a significantly higher mean MVD compared to evergreen trees. Similarly, compound-leaved trees have a higher (marginally significant) MVD than simple-leaved trees. Furthermore, we found that leaf morphology and phenology have significantly interactive effects on SL, and the compound-leafed deciduous trees exhibited the largest average SL among the four groups. There were significant correlations between the MVD and SD in all different tree groups; however, the slopes and interceptions differed within both morphology and phenology. Our results indicate that MVD, rather than SD, may be the more flexible structure for supporting the coordination between leaf water supply and demand in different leaf morphologies and phenologies. The results of the present study provide mechanistic understandings of the functional advantages of different leaf types, which may involve species fitness in community assembly and divergent responses to climate changes.

## Introduction

Forests are a crucial ecosystem with the highest biodiversity in terrestrial ecosystems, playing a vital role in soil and water conservation, carbon sequestration, and climate regulation (Trumbore et al., 2015). These processes are realized by the water adaptation strategy of trees, especially leaves. In most plants, leaves are the primary parts of photosynthetic gas exchange and light interception, which determine carbon assimilation and transpiration (Wang et al., 2022). Trees can also be classified into evergreen or deciduous trees according to their leaf phenology, and these two phenological groups often co-occur in most types of forests (Givnish, 2002). The main difference between deciduous and evergreen trees is that the former have a leafless period during the year to avoid drought stress or extremely low temperatures, whereas the latter maintains water transport to the canopy year-round (Zhang, 2012; Fu et al., 2019). It has been suggested that the two groups also differed in their carbon gain and water use strategies, with deciduous trees having greater photosynthetic capacity and higher transpiration demands, while evergreen trees had lower photosynthetic capacity but more conservative water use (Kikuzawa, 1991; Fu et al., 2012). For instance, a significant difference in stomatal density between evergreen and deciduous tree species was observed in a moist tropical forest in South Asia (Rahman et al., 2022). In contrast, another study conducted within a subtropical forest community in China reported no discernible difference in minor vein density between evergreen and deciduous species (Peng et al., 2022). Nevertheless, a limited number of studies have investigated the coordination between minor veins and stomatal densities in evergreen and deciduous tree species.

In addition, trees typically have either simple or compound leaves in terms of morphological types (Givnish, 1979). A simple leaf has a single blade unit supported by a petiole, whereas a compound leaf has multiple leaflets attached to its rachis. Two alternative hypotheses have been proposed to address whether simple leaves are homologous to compound leaves or their individual leaflets (Champagne and Sinha, 2004). A compound leaf is morphologically equivalent to a large single leaf that is divided into multiple leaflets, increasing boundary conductance and facilitating transpiration, heat dissipation, and gas exchange (Givnish, 1987; Sinha, 1997; Champagne and Sinha, 2004; Xu et al., 2009). The rachis of a compound leaf is a temporary branch and is considered more flexible and economical for coping with water stress (Givnish, 1979; Song et al., 2018; Zhao et al., 2021). However, there is currently limited research on whether this morphological difference affects the relationship between leaf water supply and demand.

Leaf veins facilitate the transportation of nutrients and water necessary for photosynthesis and transpiration processes (Sack and Scoffoni, 2013). Previous studies have shown that minor vein density is a key determinant of the leaf water supply capacity in terrestrial plants (Scoffoni et al., 2018). Higher photosynthetic rates require a faster supply of water and thus a higher minor vein density, which is coupled with higher xylem construction costs for leaf minor veins (Brodribb et al., 2007). Moreover, stomata control the gas exchange of CO_2_ and H_2_O between leaves and the atmosphere (Hetherington and Woodward, 2003). Therefore, stomatal number per leaf area (stomatal density) and size play a vital role in controlling maximum transpiration rate (which is positively related to theoretical maximum stomatal conductance (*g*_max_)) and thus, leaf water demand (Franks and Beerling, 2009). The coordination between leaf water supply and demand, indicated by the positive correlation between minor vein density and stomatal density, enables leaves to optimize photosynthetic advantages while minimizing associated costs (Brodribb and Jordan, 2011; Carins Murphy et al., 2012). This positive correlation has been demonstrated in many species (Brodribb et al., 2013; Zhang et al., 2014; Zhao et al., 2016; Wen et al., 2020). The leaf morphology (simple-leafed or compound-leafed) and phenology (evergreen or deciduous) of angiosperm tree species are the most common functional traits and are known to reflect contrasting adaptation strategies. However, the effects of these two traits and their interaction on the coordination of leaf water supply and demand remain poorly understood.

A comparison of the coordination between the leaf water supply and demand of different species under the same environmental conditions could reveal different adaptation strategies (Carins Murphy et al., 2013; Zhao et al., 2016; Zhao et al., 2020). A linear regression analysis between stomatal density and minor vein density has been used for these comparisons. For example, Carins Murphy et al (2012; 2013). found a correlation between stomatal density and minor vein density in *Toona ciliata* M. Roem., which did not change in leaves produced by plants acclimated to different vapor pressures and irradiance treatments. In addition, Zhao et al. (2017) suggested that the stomatal number per minor vein length (SV) may be a key trait associated with leaf water supply and demand. Zhao et al. (2017) also found that three leguminous species under the same light conditions exhibited a stable stomatal number per minor vein length, indicating coordination between leaf water supply and demand. Furthermore, when the environmental conditions changed, the stomatal number per minor vein length changed accordingly. Therefore, as a new functional trait, stomatal number per minor vein length needs to be studied further in different species, especially in angiosperm trees with different leaf morphologies and phenologies.

In summary, both compound leaves and deciduous phenology can provide advantages to trees adapted to drought stress (Zhao et al., 2022); sometimes both of them will appear on the same tree species at the same time, and traditional studies that classify trees based on only one feature (leaf morphology or phenology) are inadequate (Fu et al., 2019; Zhao et al., 2019; Wang et al., 2020). In the present study, we classified tree species based on their leaf morphology and phenology, including simple-leafed evergreen trees, simple-leafed deciduous trees, compound-leafed evergreen trees, and compound-leafed deciduous trees. We hypothesized that tree species with compound leaves and deciduous habits would exhibit higher minor vein density, xylem construction costs of leaf minor veins, theoretical maximum stomatal conductance, stomatal density, and stomatal number per minor vein length due to their superior water-use efficiency compared to their simple and evergreen counterparts (Xu et al., 2009; Zhang, 2012). Moreover, we proposed that to optimize photosynthetic yield, a correlation between minor vein density and stomatal density would exist in angiosperm trees, independent of their leaf morphology and phenology.

## Materials and methods

### Site and sampling

The sampling area of the study was located in the southern part of China (Guangdong province, 23°4′–24°26′ N, 110°9′–116°34′ E), which is dominated by subtropical evergreen broad-leaved forests with a proportion of deciduous tree species (Zhou et al., 2014). The annual mean temperature is 20.9°C, and the annual mean precipitation is 1,841.8 mm with nearly 80% occurring in the wet season (April to September).

In the core area of natural protected forests, trees are distributed randomly and grown in their natural state with less human disturbance. Trees with a diameter at breast height of >5 cm were chosen from one plot (5 ha). For each tree species, three to four individuals were chosen, and approximately three to five sunlit leaves per individual were collected. In total, the leaves of 108 tree species were collected (**Supplemental Data 1**), including 83 simple-leafed species, 25 compound-leafed species, 78 evergreen species, and 30 deciduous species in accordance with the categorization of the description in Flora of China. We further separated these into additional four groups: compound-leafed deciduous trees (CLD), compound-leafed evergreen trees (CLE), simple-leafed deciduous trees (SLD), and simple-leafed evergreen trees (SLE). The leaves of three CLD, two CLE, three SLD, and eight SLE were obtained from one or two individuals.

### Measurement of leaf traits

The collected leaves were scanned (HP Scanjet G3110, Hewlett-Packard Development Co., Palo Alto, CA) to obtain images of the leaves. We then used Image J (http://rsbweb.nih.gov/ij/index.html) to measure the leaf area. Leaf samples were stored in 70% alcohol for further analysis.

Stomatal density, length (SL), and width (SW) were determined from the abaxial cuticles of the leaves by the impression method (Zhao et al., 2016, 2017). We applied clear nail varnish to a 1-cm^2^ patch on the middle part of the leaf surface. After 3 min, the nail polish was removed and mounted on a glass slide to be observed under a microscope (LEICA DM 2500, Germany). The stomata images were taken under ×200 or ×400 magnification (ca. 20 stomata in the field).

To investigate the demand for water produced by stomatal size and density, we calculated the *g*_max_ based on the measured stomatal anatomy (Waggoner, 1970; Franks and Farquhar, 2001; Brodribb and Jordan, 2011). The *g*_max_ was estimated using the following equation:


(1) $$g_{\text{max}}=\frac{d}{v}\times D\times \frac{a}{l+\frac{\pi }{2}\sqrt{a/\pi }}$$

where *d* is the diffusivity of water in air (24.9 × 10^−6^ m^2^ s^−1^, 25°C), *υ* is the molar volume of air (24.4 × 10^−3^ m^3^ mol^−1^, 25°C, 101.3 kPa), *D* is the stomatal density, *a* is the maximum pore area, and *l* is the pore depth that is represented by mean stomatal width; the maximum pore area was calculated from the stomatal length (Brodribb et al., 2013).

Leaves utilized for stomatal trait measurements were also employed for assessing minor vein density. These leaves were placed in glass tubes containing 5% NaOH aqueous solution and heated in a water bath (Yiheng, HWS24, China) until the veins were exposed. Subsequently, the leaves were soaked in distilled water for 30 min, dyed with a 1% methylene blue solution, rinsed again, mounted on slides, and photographed using a microscope equipped with a digital camera (LEICA DM 2500, Germany). We measured the minor vein density and thickness (MVT) using Image J. SV (No. mm^−1^) was calculated by dividing the stomatal density by the minor vein density.

The xylem construction cost (CC) was calculated using vein density and vein diameter, which reflect the difference in leaf vein investment among varying angiosperm tree groups (Mckown et al., 2010). We estimated the xylem construction costs of leaf minor veins with a dimensionless index of cell wall volume per leaf area (CC = π × MVT × MVD; Mckown et al., 2010; Schneider et al., 2017).

### Data analyses

To determine the influence of leaf phenology, leaf morphology, and their interaction on all nine leaf traits, we employed a nonparametric test (Scheirer–Ray–Hare test) due to the non-normal distribution of certain leaf traits (e.g., LA, SD, *g*_max_, MVD, and SV; **Supplementary Figure S1**; **Supplementary Table S1**) even after data transformation. Distinct patterns and variations in functional traits and tree species were further explored separately via nonmetric multidimensional scaling (NMDS). To assess the relationships between minor vein density and stomatal density, standardized major axis (SMA) regression was utilized, having log-transformed both minor vein density and stomatal density prior to the analysis. The assumptions of the SMA regression, which included checking the independence of residuals, linearity of minor vein density and stomatal density, and equal variance of residuals, were checked using the residual plot (fitted values vs. residuals) (Warton et al., 2006). The normality of residuals was determined using the quantile–quantile plot. From the residual plots and normal Q–Q plot, we can see that all assumptions of the SMA regression were met (**Supplementary Figures S2**-**S4**). Statistical analyses were conducted with SPSS 16.0 (SPSS Inc., IBM, Armonk, NY, USA) and R ver. 4.0.2 (R Core Team, 2020). Species mean values were used to carry out all the above statistical analyses.

## Results

All 108 tree species investigated in this study were hypostomatic. There were no significant differences in the nine leaf traits between the simple- and compound-leafed trees, and only leaf MVD and CC were marginally affected by leaf morphology (**Tables 1**, **2**). Similarly, there were no significant differences in LA, SD, stomatal length, stomatal width, maximum modeled *g*_max_, or CC between evergreen and deciduous trees (**Table 1**). However, the evergreen trees had significantly lower mean MVD but higher MVT and SV than the deciduous trees (**Table 1**). We also found that SL was significantly affected by the interaction between leaf morphology and leaf phenology (**Table 2**). In simple leaf trees, the SL of deciduous trees (18.2 ± 0.8 μm) was 8.9% lower than that of evergreen trees (19.8 ± 0.5 μm), whereas in compound leaf trees, the SL of deciduous trees (20.8 ± 1.1 μm) was 13.5% higher than that of evergreen trees (18.0 ± 1.0 μm) (**Table 3**).

**Table 1:** Leaf functional traits (mean ± standard error) of 108 angiosperm trees with different leaf morphologies and phenologies.

Traits	Units	Leaf morphology		Leaf phenology	
		Simple-leafed trees (83)	Compound-leafed trees (25)	Evergreen trees (78)	Deciduous trees (30)
LA	cm^2^	43.7 ± 4.2	35.1 ± 7.6^ns^	39.0 ± 3.8	48.9 ± 7.5^ns^
SD	No. mm^−2^	335 ± 14	357 ± 25^ns^	338 ± 15	345 ± 24^ns^
SL	μm	19.5 ± 0.4	19.6 ± 0.8^ns^	19.5 ± 0.4	19.4 ± 0.7^ns^
SW	μm	13.8 ± 0.4	14.3 ± 0.8^ns^	14.1 ± 0.4	13.4 ± 0.7^ns^
*g*_max_	μmol H_2_O m^−2^s^−1^	0.46 ± 0.02	0.48 ± 0.03^ns^	0.46 ± 0.02	0.48 ± 0.02^ns^
MVD	mm mm^−2^	7.10 ± 0.23	9.90 ± 0.44^ns^	7.10 ± 0.3	9.60 ± 0.70^**^
MVT	μm	21.4 ± 0.5	18.5 ± 0.9^ns^	21.9 ± 0.5	17.8 ± 0.7^***^
SV	No. mm^−1^	48.3 ± 1.9	39.0 ± 3.6^ns^	49.2 ± 2.0	38.2 ± 2.4^*^
CC	–	0.47 ± 0.01	0.53 ± 0.02^ns^	0.47 ± 0.01	0.50 ± 0.02^ns^

^*^p< 0.05; ^**^p< 0.01; ^***^p< 0.001; ns, p > 0.05—significant difference.

LA, leaf area; SD, stomatal density; SL, stomatal length; SW, stomatal width; g_max_, maximum modeled stomatal conductance; MVD, minor vein density; MVT, minor vein thickness; SV, stomatal number per minor vein length; CC, construction cost of minor vein network per leaf area.

**Table 2:** The impact of leaf morphology, leaf phenology, and their interaction on leaf traits in 108 angiosperm trees. .

Traits	Leaf morphology		Leaf phenology		Leaf phenology × morphology	
	*H*	*p*-value	*H*	*p*-value	*H*	*p*-value
LA	1.33	ns	1.52	ns	2.60	ns
SD	0.06	ns	0.05	ns	1.50	ns
SL	0.00	ns	0.01	ns	5.62	–^*^
SW	1.08	ns	1.24	ns	1.71	ns
*g*_max_	0.01	ns	1.28	ns	0.08	ns
MVD	3.41	0.06	9.04	–^**^	2.13	ns
MVT	1.86	ns	13.18	–^***^	2.65	ns
SV	2.16	ns	5.08	–^*^	0.11	ns
CC	3.13	0.08	0.56	ns	0.23	ns

^*^p< 0.05; ^**^p< 0.01; ^***^p< 0.001; ns, p > 0.05—H statistics (H) and statistical significance (p-values).

LA, leaf area; SD, stomatal density; SL, stomatal length; SW, stomatal width; g_max_, maximum modeled stomatal conductance; MVD, minor vein density; MVT, minor vein thickness; SV, stomatal number per minor vein length; CC, construction cost of minor vein network per leaf area.

**Table 3:** Leaf functional traits for evergreen and deciduous trees and for compound- and simple-leaved trees.

Traits	Units	Simple-leafed trees		Compound-leafed trees	
		Evergreen (67)	Deciduous (16)	Evergreen (11)	Deciduous (14)
LA	cm^2^	39.3 ± 4.2	62.4 ± 11.7	37.4 ± 7.7	33.4 ± 7.6
SD	No. mm^−2^	330 ± 16	353 ± 26	382 ± 48	337 ± 42
SL	μm	19.8 ± 0.5	18.2 ± 0.8	18.0 ± 1.0	20.8 ± 1.1
SW	μm	14.1 ± 0.48	12.3 ± 0.7	13.9 ± 1.2	14.6 ± 1.3
*g*_max_	μmol H_2_O m^−2^s^−1^	0.46 ± 0.02	0.47 ± 0.02	0.46 ± 0.05	0.49 ± 0.04
MVD	mm mm^−2^	6.76 ± 0.24	8.72 ± 0.42	8.9 ± 0.92	10.69 ± 1.33
MVT	μm	22.2 ± 0.5	18.1 ± 0.7	19.7 ± 0.7	17.5 ± 1.3
SV	No. mm^−1^	50.1 ± 2.3	40.4 ± 2.2	43.5 ± 3.4	35.6 ± 4.5
CC		0.46 ± 0.01	0.49 ± 0.02	0.53 ± 0.04	0.52 ± 0.03

Data are mean ± SE.

LA, leaf area; SD, stomatal density; SL, stomatal length; SW, stomatal width; g_max_, maximum modeled stomatal conductance; MVD, minor vein density; MVT, minor vein thickness; SV, stomatal number per minor vein length; CC, construction cost of minor vein network per leaf area.

**Figure 1:**
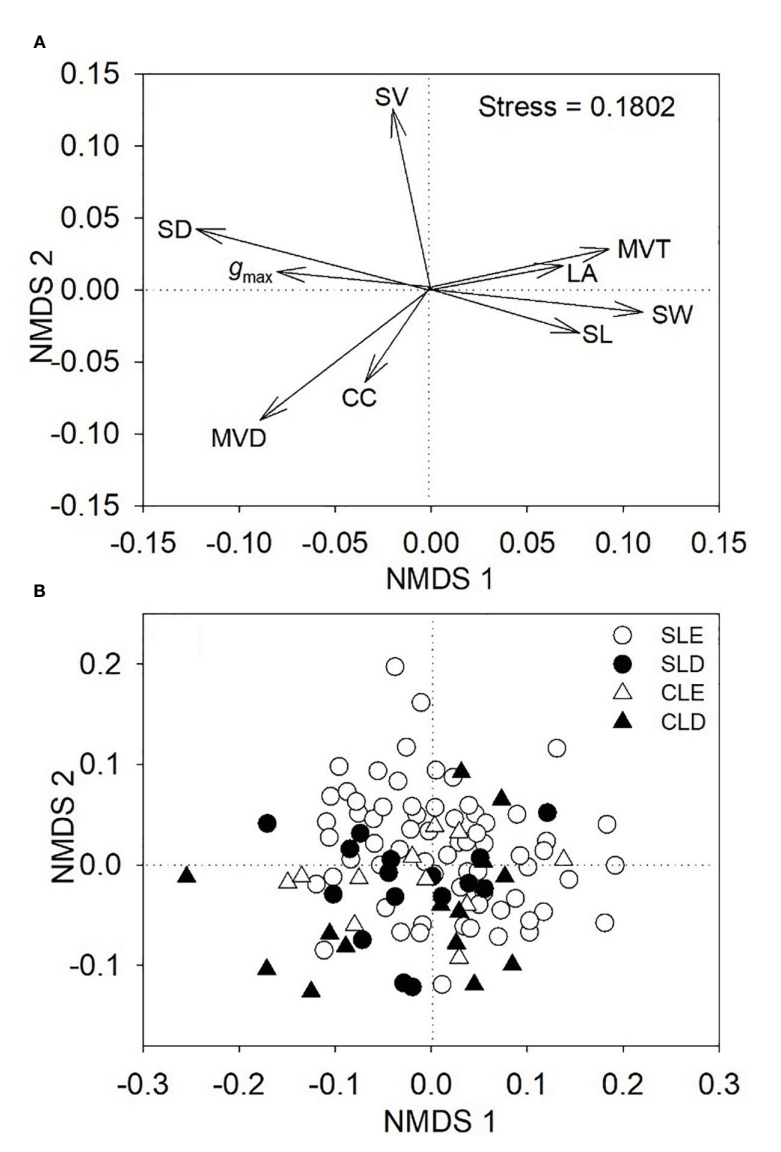
The first two axes of nonmetric multidimensional scaling (NMDS) ordination for nine functional traits **(A)** and 108 tree species **(B)** with different leaf morphologies and phenologies. CLD, compound-leafed deciduous trees; CLE, compound-leafed evergreen trees; SLD, simple-leafed deciduous trees; SLE, simple-leafed evergreen trees.

The stress value (0.1802) indicated that the NMDS analysis captured the information of the high-dimensional space more effectively (**Figure 1**). NMDS 1 was loaded with SD, *g*_max_, and MVD on the negative side and SL, SW, and MVT on the positive side, whereas NMDS 2 was loaded with SV on the positive side (**Figure 1**).

**Figure 2:**
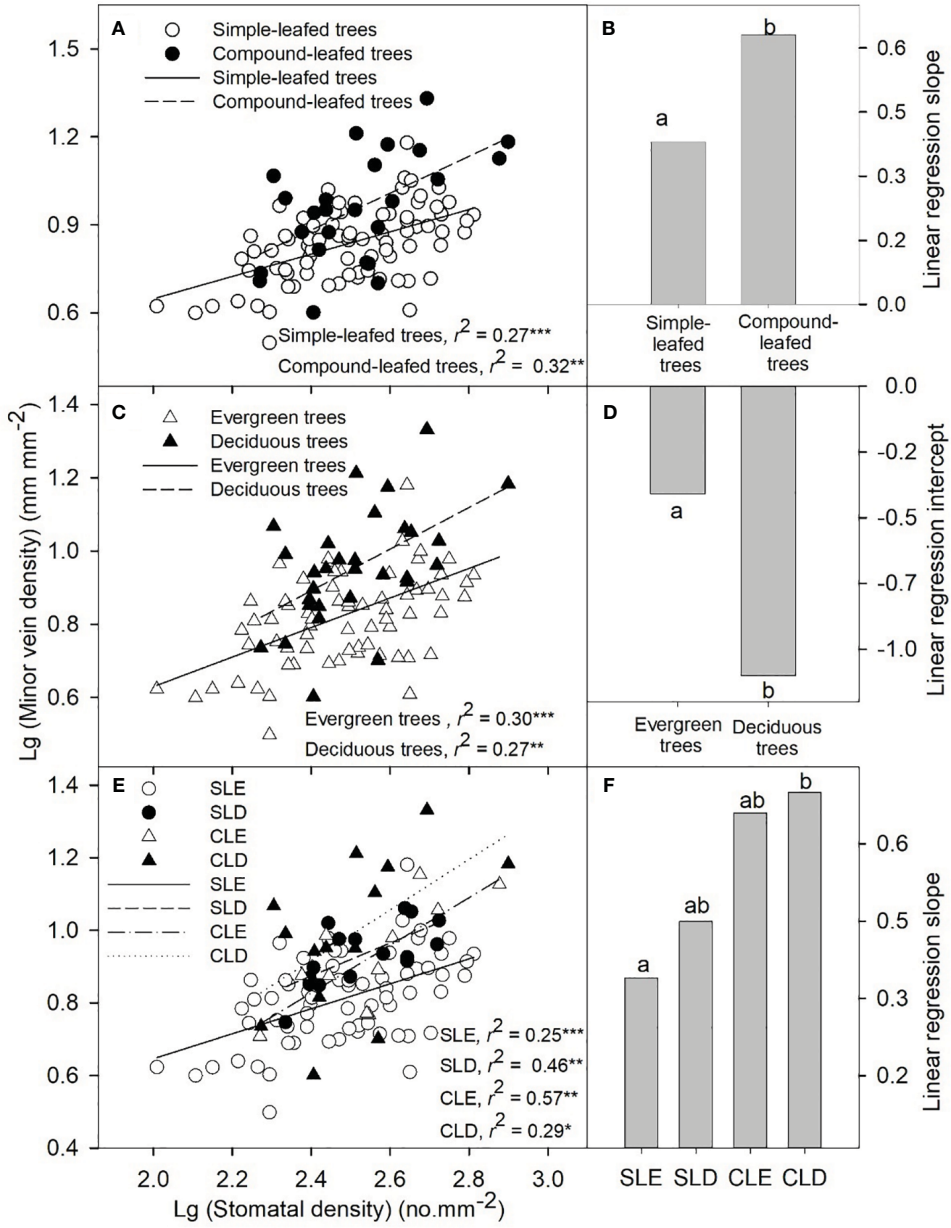
Correlations between stomatal density and minor vein density of angiosperm trees with different leaf morphologies and phenologies **(A, C, E)**. Different lowercase letters in each panel indicate significant differences of linear slope or intercept among tree groups **(B, D, F)**. Each symbol represents one species. *p< 0.05; **p< 0.01; ***p< 0.001. SLE, simple-leafed evergreen trees; SLD, simple-leafed deciduous trees; CLE, compound-leafed evergreen trees; CLD, compound-leafed deciduous trees.

A significant positive correlation was found between MVD and SD in simple-leafed trees (*r* ^2^ = 0.27, *p*< 0.001) and compound-leafed trees (*r* ^2^ = 0.32, *p*< 0.01) (**Figure 2A**). The regression slope for compound-leafed trees was significantly higher than that for simple-leafed trees (**Figure 2B**). Similarly, significant correlations were found between MVD and SD in both evergreen and deciduous trees (evergreen trees: *r* ^2^ = 0.30, *p*< 0.001; deciduous trees: *r* ^2^ = 0.27, *p*< 0.01; **Figure 2C**). The regression intercept for evergreen trees was significantly higher than that for deciduous trees (**Figure 2D**). When the 108 species were separated into four groups based on leaf morphology and phenology, we found a significant positive correlation between MVD and SD in each group (simple-leafed, evergreen trees: *r* ^2^ = 0.25, *p*< 0.001; simple-leafed, deciduous trees: *r* ^2^ = 0.46, *p*< 0.01; compound-leafed, evergreen trees: *r* ^2^ = 0.57, *p*< 0.01; compound-leafed, deciduous trees: *r* ^2^ = 0.29, *p*< 0.05; **Figure 2E**). The linear regression slopes of simple-leafed and evergreen trees were significantly lower than those of compound-leafed and deciduous trees (**Figure 2F**).

## Discussion

The present study investigated the effect of leaf phenology on leaf vein characteristics and found that deciduous trees had significantly higher minor vein density, lower minor vein thickness, and lower stomatal number per minor vein length, partially supporting our first hypothesis. The results were consistent with a meta-analysis of vein density in 796 species, in which deciduous species had a higher mean vein density than evergreens within given biomes and growth forms (Sack and Scoffoni, 2013). Water transport efficiency is much higher in small veins than diffusion efficiency between cells (Brodribb et al., 2007; Sack and Scoffoni, 2013). Leaf vein traits are commonly considered an indicator of water supply capacity (Brodribb et al., 2007; Carins Murphy et al., 2013), and our study suggested that deciduous trees may have higher water supply efficiency than evergreen trees. Leaves with a higher minor vein density are better equipped to meet the water demand of stomata, resulting in a higher stomatal conductance and photosynthetic rate (Brodribb and Jordan, 2011). Our findings suggest that deciduous tree species, despite their shorter growth cycle and leaf lifespan, can gain sufficient carbon assimilation with their higher leaf water transport capacity in a shorter period than evergreen trees. However, we did not find significant differences in either the xylem construction cost of leaf minor veins or the theoretical maximum stomatal conductance between evergreen and deciduous tree species. Deciduous tree species had a higher minor vein density but a lower minor vein thickness, resulting in similar construction costs compared to evergreen tree species (**Table 1**). The modeled *g*_max_ was calculated based on the values of stomatal density, stomal length, and stomatal width. Since none of these three traits differed between evergreen and deciduous tree species, *g*_max_ was also similar between these two groups (**Table 1**). However, deciduous tree species are more likely to have higher actual stomatal conductance due to their higher leaf water transport capacity.

Contrary to our initial hypothesis, we observed no significant differences in stomatal or leaf minor vein characteristics between compound- and simple-leafed tree species. Although compound-leafed tree species tend to exhibit higher minor vein density and construction costs compared to simple-leaved tree species, the difference was not statistically significant (**Table 2**). In contrast, Yang et al. (2019) demonstrated that compound-leafed tree species in temperate forests possess significantly higher stem hydraulic efficiency and photosynthetic rates than simple-leafed trees. This indicates that differences in stem hydraulic efficiency and photosynthetic rates among varying leaf morphologies also depend on wood anatomy types. The similarity in leaf vein and stomatal traits observed in this study might be attributed to the fact that most tree species in subtropical and tropical regions belong to the diffuse-porous type. Furthermore, compound-leafed trees are better adapted to seasonal drought stress than simple-leafed tree species (Yang et al., 2019). Therefore, the well-watered conditions at the study site might have contributed to the similar leaf traits between the two groups.

Interestingly, we found that there were significant interaction effects of leaf morphology and leaf phenology on stomatal length in the present study. Stomatal length is an important trait that determines the size of the aperture for stomata; species with a larger stomatal size tend to have greater potential stomatal conductance (Hetherington and Woodward, 2003; Brodribb et al., 2013). The compound-leafed deciduous tree species with the highest stomatal length, as well as the highest minor vein density, are potentially more likely to have greater stomatal conductance and photosynthetic rate (Brodribb et al., 2007). Our study provided valuable insights into how variations in leaf morphology and phenology can influence important anatomical traits, and further research on the interaction effect of leaf morphology and phenology on plant physiological and ecological function is needed.

Despite the significant effects of leaf phenology on minor vein density and the interaction between leaf phenology and morphology on stomatal length, we observed coordination between stomatal density and minor vein density in each group (**Figure 2**). The observed relationships between leaf morphology and anatomy may be related to the hypothesis suggesting coordination between water supply and demand (Brodribb and Jordan, 2011; Sun et al., 2014; Zhao et al., 2017). Similarly, coordination between minor vein density and stomatal density was found in both tropical and subtropical mountain forests, even though tree species in these two forests differed in minor vein density and exhibited similar stomatal density (Zhao et al., 2016). We found that leaf phenology and morphology led to significant changes in the correlation between stomatal density and minor vein density, as evidenced by differences in regression slope and intercept among various groups (**Figure 2**). A significantly higher linear regression slope or intercept indicates that compound-leafed and deciduous tree species tend to have higher minor vein density compared to simple-leafed and evergreen tree species at the same stomatal density. This redundancy of minor veins enables compound-leafed and deciduous tree species to not only achieve higher hydraulic efficiency but also exhibit better tolerance to water deficits caused by seasonal drought and high temperatures at noon (Sack and Scoffoni, 2013).

## Conclusion

In conclusion, our results reveal that leaf phenology significantly affects leaf minor vein density, minor vein thickness, and stomatal number per minor vein length, while leaf morphology has no significant impact on either leaf vein or stomatal characteristics. Our findings also demonstrate that stomatal length is significantly affected by the interaction between leaf morphology and phenology, with compound-leafed deciduous tree species displaying the highest stomatal length and potentially greater stomatal conductance during the warm and wet rainy seasons. Coordination between leaf minor vein density and stomatal density exists among various phenological and leaf-form groups. Our study provides mechanistic insights into the functional advantages of different leaf types and may shed light on community assembly and the divergent responses of tree species from different functional groups to climate changes in subtropical forests.

## Data availability statement

The original contributions presented in the study are included in the article/**Supplementary Material**. Further inquiries can be directed to the corresponding authors.

## Author contributions

WZ and PZ conceived and designed the research. WZ, QM, GL, and YL conducted experiments. WZ and PF analyzed the data. WZ wrote the manuscript. PZ, PF, and JX edited this manuscript. All authors read and approved the manuscript.
