# Supplementary material for: Effect of leaf phenology and morphology on the coordination between stomatal and minor vein densities
Source: Front Plant Sci. 2023 Jul 26;14:1051692. doi: 10.3389/fpls.2023.1051692 (PMC10411352; doi:10.3389/fpls.2023.1051692)
Supplement: Supplementary file 1 [file DataSheet_1.docx]

**Supplemental data 2**

**Table S1** The Skewness, Kurtosis and P value of the Jarque-Bera test. The *P* values of the Jarque-Bera test that significantly lower than 0.05 were indicated in bold.

| Trait | Skewness | Kurtosis | *P values* |
| --- | --- | --- | --- |
| LA | 1.64 | 5.52 | ***<* 0.001** |
| SD | 0.86 | 3.72 | ***<* 0.001** |
| SL | 0.44 | 3.64 | 0.088 |
| SW | 0.38 | 2.96 | 0.26 |
| *g_max_* | 1.04 | 4.05 | ***<* 0.001** |
| MVD | 1.58 | 6.87 | ***<* 0.001** |
| MVT | -0.01 | 3.22 | 0.89 |
| SV | 0.96 | 3.95 | ***<* 0.001** |
| CC | 0.09 | 2.67 | 0.73 |

LA = Leaf area, SD = Stomatal density, SL = Stomatal length, SW = Stomatal width, gmax = Maximum modelled stomatal conductance, MVD = Minor vein density, MVT = Minor vein thickness, SV = Stomatal number per minor vein length, CC = Construction cost of minor vein network per leaf area.


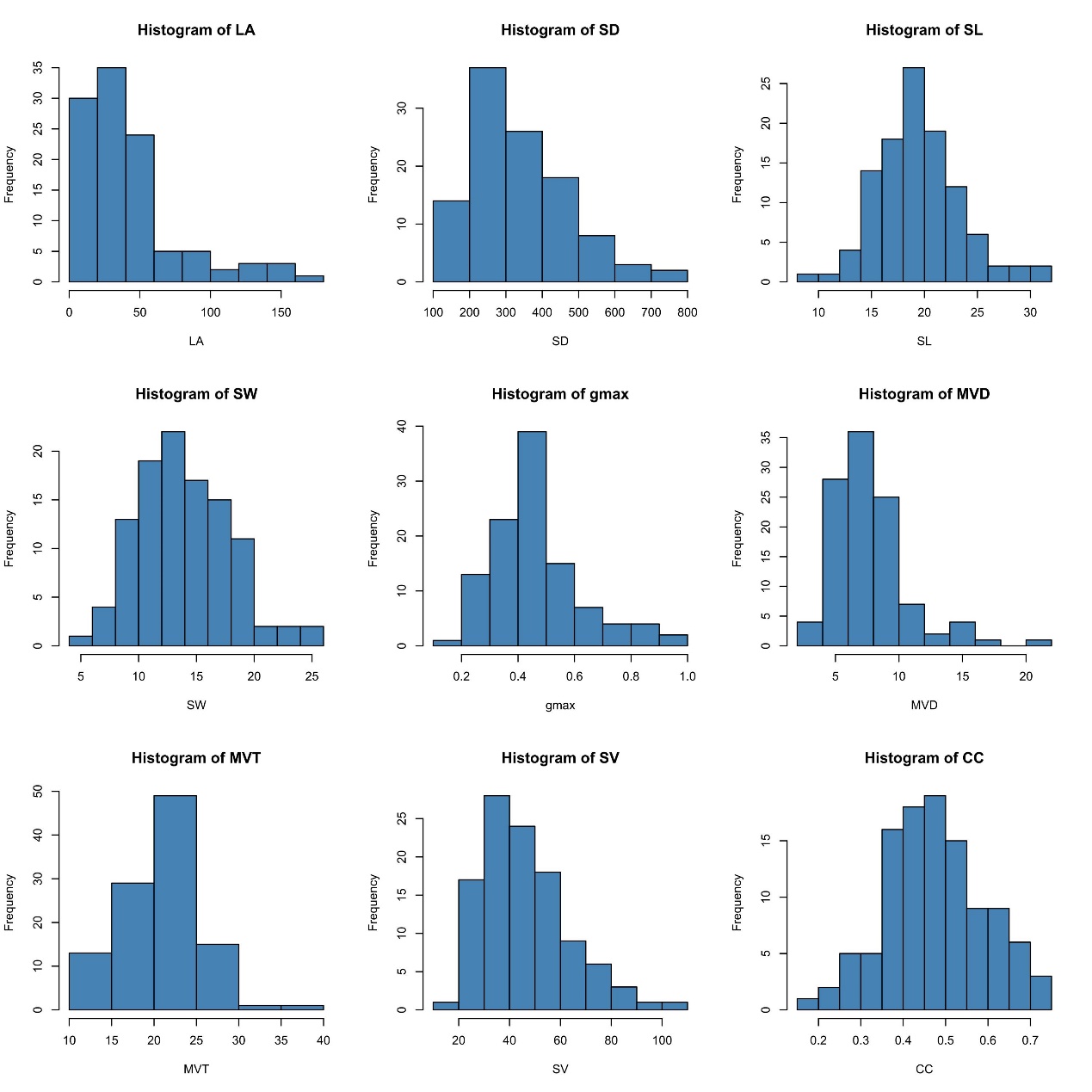


**Fig. S1** Histogram of the nine studied traits. LA = Leaf area, SD = Stomatal density, SL = Stomatal length, SW = Stomatal width, *g*_max_ = Maximum modelled stomatal conductance, MVD = Minor vein density, MVT = Minor vein thickness, SV = Stomatal number per minor vein length, CC = Construction cost of minor vein network per leaf area.


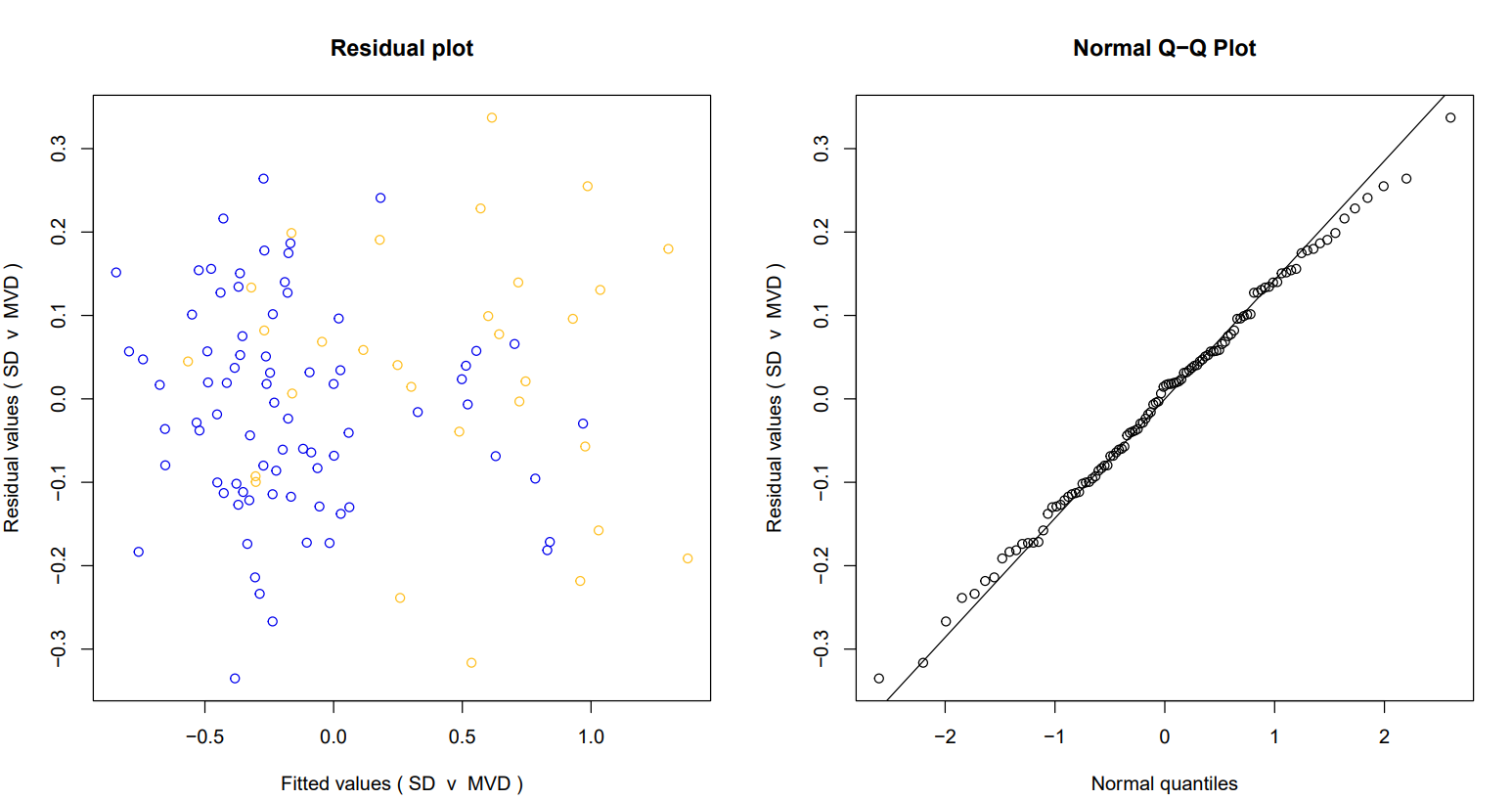


**Fig. S2** Residuals plot and Q-Q plot of the standardized major axis regression of stomatal density vs. minor vein density for evergreen and deciduous tree species.


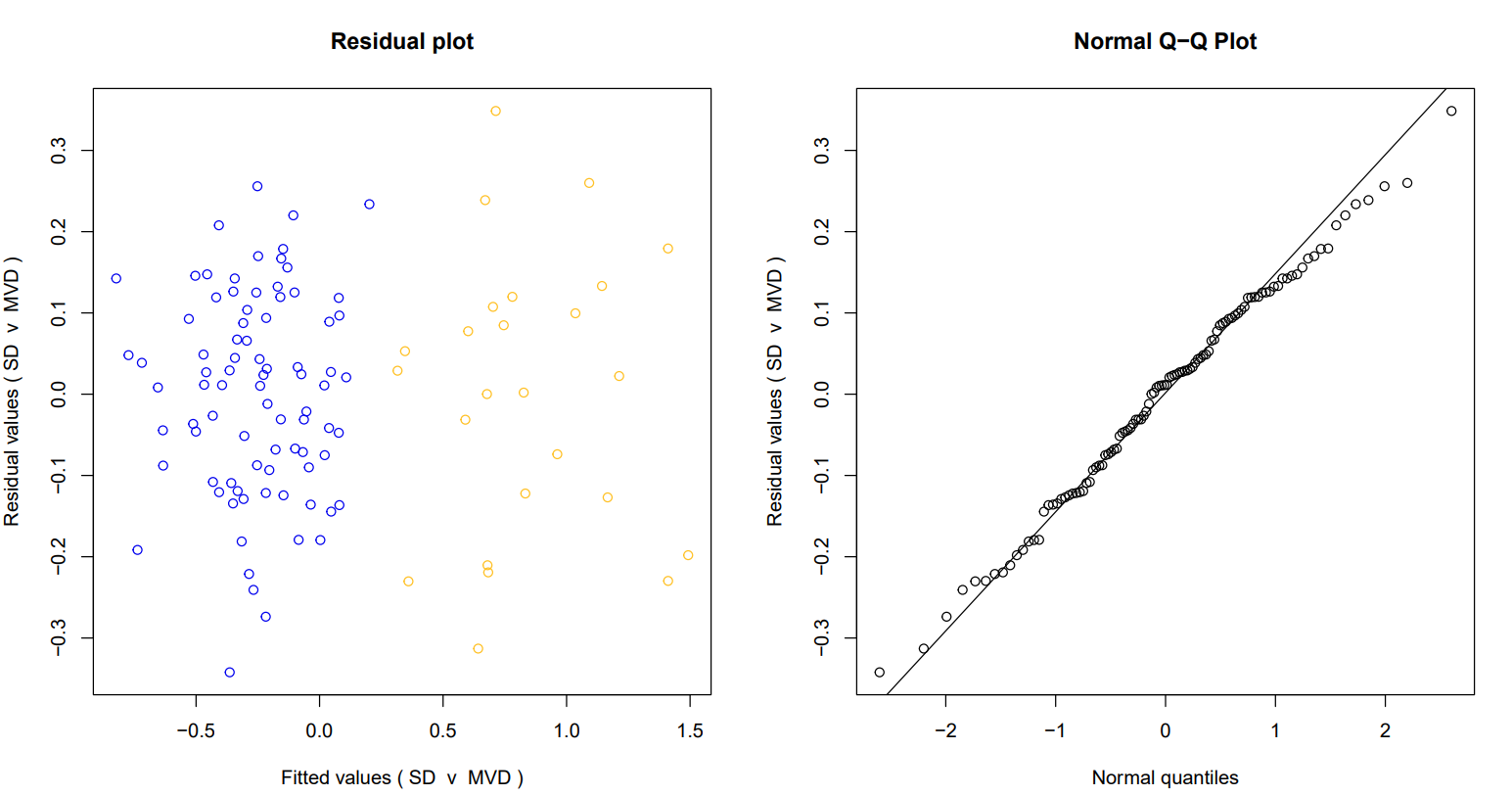


**Fig. S3** Residuals plot and Q-Q plot of the standardized major axis regression of stomatal density vs. minor vein density for simple-leafed and compound-leafed tree species.


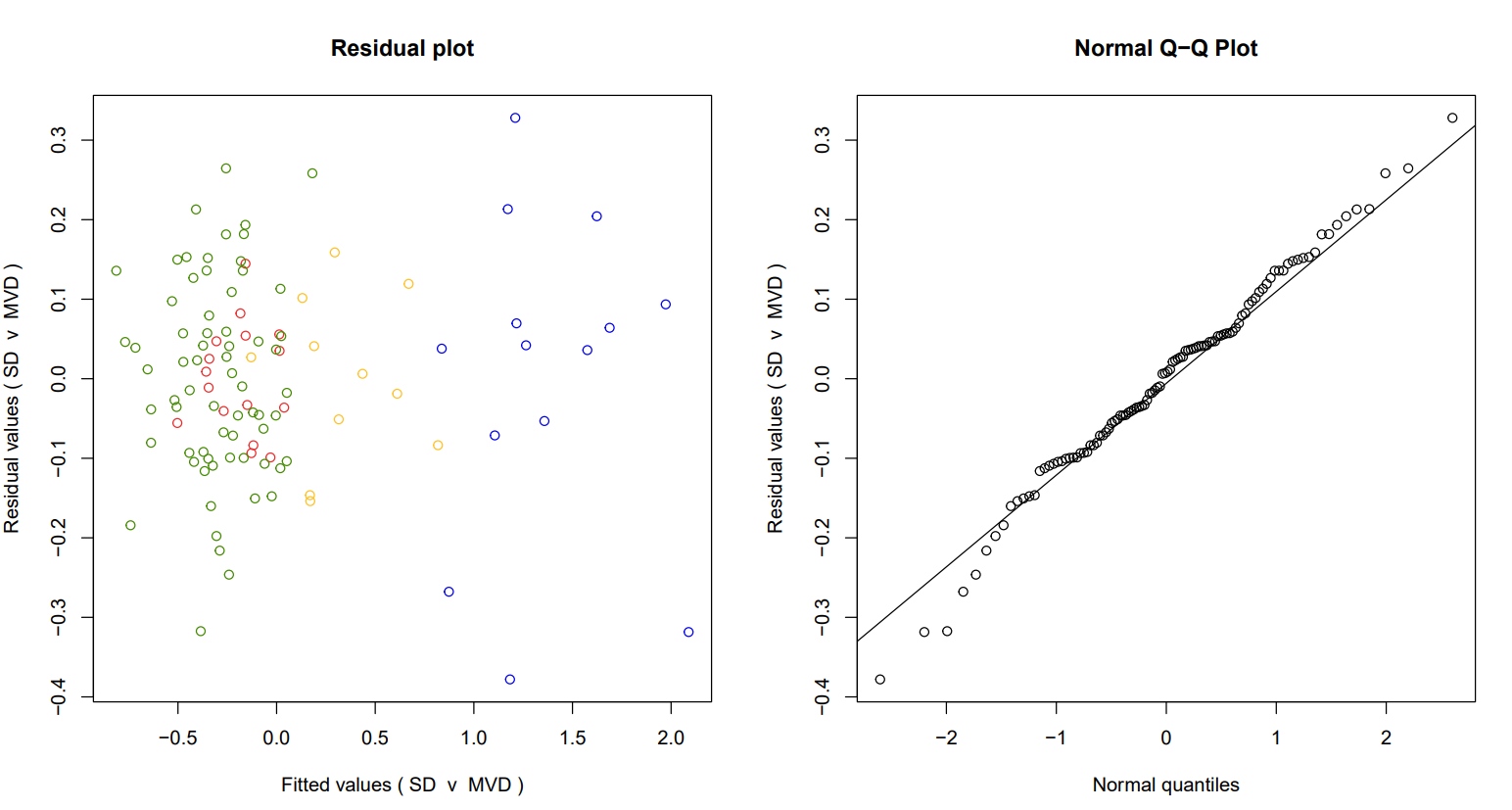


**Fig. S4** Residuals plot and Q-Q plot of the standardized major axis regression of stomatal density vs. minor vein density for simple-leafed evergreen trees species, simple-leafed deciduous trees, compound-leafed evergreen trees species, compound-leafed deciduous tree species and compound-leafed tree species.
